# Hermansky-Pudlak Syndrome: A Rare Congenital Disorder With Interstitial Lung Disease

**DOI:** 10.7759/cureus.65035

**Published:** 2024-07-21

**Authors:** Sreeja Sanampudi, Aashna Vajramani, Kiran Batra

**Affiliations:** 1 Radiology, University of Texas (UT) Southwestern Medical Center, Dallas, USA; 2 Radiology, Texas Agricultural and Mechanical (A&M) University, College Station, USA; 3 Cardiothoracic Imaging, University of Texas (UT) Southwestern Medical Center, Dallas, USA

**Keywords:** ild interstitial lung disease, honeycombing, traction bronchiectasis, ground-glass opacities, pulmonary interstitial fibrosis

## Abstract

Hermansky-Pudlak syndrome (HPS) is a genetic multisystemic disorder with oculocutaneous albinism, granulomatous colitis, bleeding diathesis, and pulmonary fibrosis. Multiple subtypes of HPS exist, with certain types having higher predilection for pulmonary fibrosis. This case report focuses on the demonstration of pulmonary imaging findings seen in a patient. Several imaging features overlap with idiopathic pulmonary fibrosis including traction bronchiectasis, pleural and peribronchovascular thickening, and reticulations. This case report highlights the differences seen in lung disease associated with HPS compared to other interstitial lung diseases, in addition to the multi-systemic features of HPS.

## Introduction

Hermansky-Pudlak syndrome (HPS) is an autosomal recessive multisystemic disorder that is associated with oculocutaneous albinism, pulmonary fibrosis, granulomatous colitis, and bleeding diathesis. It is caused by mutations in the HPS gene with different subtypes resulting in different clinical manifestations [1]. There are ten known subtypes affecting people of various ethnic origins including, European, Japanese, Indian, Middle Eastern, Chinese, and non-Puerto Rican Hispanics [1]. All patients regardless of the subtype have oculocutaneous albinism and bleeding diathesis [1]. In this case report, we discuss the primary clinical and imaging findings in patients with HPS in addition to diagnostic imaging criteria based on high-resolution computed tomography (HRCT). 

## Case presentation

A 33-year-old white male with a history of inflammatory bowel disease (IBD) status post total colectomy, known diagnosis of HPS made at the time of diagnosing IBD, multiple episodes of acute kidney injury, bleeding diathesis, plasma dysfunction presented with worsening dyspnea and cough at age 30 and was initially treated for pneumonia and provoked deep venous thrombosis. He has since had recurrent DVTs requiring long-term anticoagulation. Although the acute dyspnea had resolved, the patient had a progressive decline in pulmonary function requiring continuous oxygen initially 1-2 L to 15 L of O2 at the time of his last hospital admission. The physical exam was positive for oculocutaneous albinism and crackles at lung bases. Labs indicated platelets of 143, creatinine of 2.1 mg/dL, protime of 43.9 seconds, international normalized ratio of 4.2, and partial thromboplastin time of 38.2 seconds.

**Figure 1:**
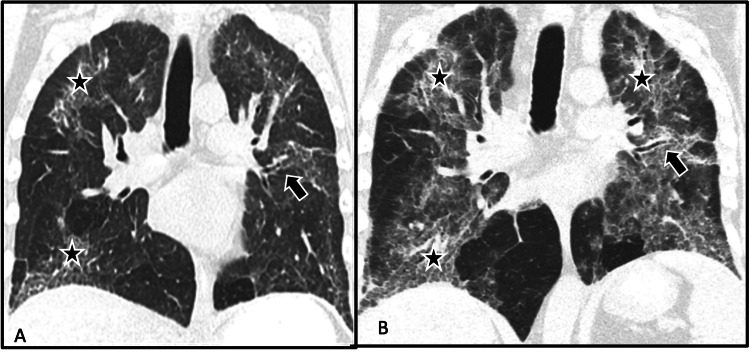
Progression of disease. (A) Coronal CT image at baseline showing few early ground glass reticular densities in the periphery predominantly at the lung apices and lung bases (black star) with mild traction bronchiectasis (black arrow). (B) Coronal CT image two years later when the patient presented with worsening dyspnea. The image shows the progression of lung disease with progression in the distribution of ground glass and reticular densities (black star) now involving the central regions in the upper and mid lung bilaterally with signs of fibrosis and mild traction bronchiectasis (black arrow).

Chest imaging had progressively declined during hospitalization. Initial chest CT following treatment of pneumonia demonstrated ground glass opacities throughout both lungs with early signs of established pulmonary fibrosis with irregular interlobular septal thickening and intralobular reticular opacities (Figure 1A, 1B).

**Figure 2:**
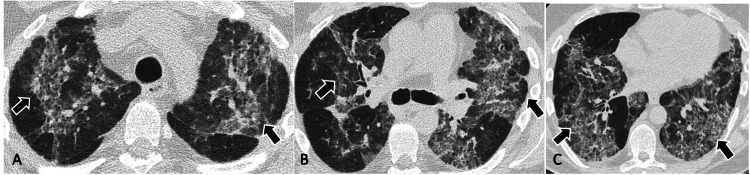
High-resolution CT performed two years after the initial scan. High-resolution axial CT at the upper lobes (A), carina (B), and lung bases (C) demonstrating ground glass opacities and findings of fibrosis with areas of traction bronchiectasis, overall encompassing more than two-thirds of the lungs (black arrows).

Subsequent imaging during the last hospitalization demonstrated worsening of these ground glass opacities with new subpleural reticulations, additional new ground glass and consolidative opacities bilaterally, traction bronchiectasis, air trapping, and overall worsening of fibrosis (Figures 2A, 2B, 2C).

He had end-stage HPS-associated interstitial lung disease (ILD) and was unable to receive a lung transplant due to the poor quality of the donor's lungs, and later expired due to respiratory failure.

## Discussion

HPS is caused by genetic mutations that result in improper functioning of lysosomal-related organelles [2]. The gold standard test for diagnosis is identifying the absence of dense bodies on electron microscopy [2]. There are multiple subtypes of HPS. HPS-1, HPS-2, and HPS-4 have been associated with pulmonary fibrosis with HPS-1 having the highest mortality rates and is considered a more severe form of the disease [3,4]. In HPS-1 patients, imaging findings typically manifest in patients 30 years and older with none to minimal changes identified in patients less than 20 years of age [5]. Additional subtypes often have milder symptoms and can have slightly varied clinical presentations; for example, HPS-10 has been associated with neurological symptoms [6]. HPS gene mutations result in changes in intracellular signaling and downstream activation of various enzymes and molecules via defective lysosome-related organelles and biogenesis of lysosome-related organelle complexes [6]. Over time accumulation of ceroids which are lipid-protein complexes, results in tissue inflammation and multiorgan symptomatology associated with HPS. 

HPS-related pulmonary fibrosis and idiopathic pulmonary fibrosis are considered similar entities by the American Thoracic Society and European Respiratory Society [6]. Although the causes of these disease processes and age at presentation are different, they have similar histological features and similar clinical course [6]. The pathogenesis of pulmonary fibrosis involves the accumulation of defective lysosome-related organelles containing surfactant in type two alveolar epithelial cells [2,7]. Also, ceroid accumulation within alveolar macrophages has been associated with HPS [7]. 

Patients typically present with progressively worsening dyspnea and chronic cough. Additional symptoms include recurrent infections, pneumothorax, and neutropenia [7]. Like our patient, typical clinical manifestations include oculocutaneous albinism, ecchymoses from bleeding diathesis, and coarse crackles in the lung bases. Additionally, acrocyanosis and digital clubbing can be seen as secondary features of severe lung disease [7,8]. Pulmonary function tests often demonstrate worsening restriction with worsening fibrotic changes with a reduction in the diffusing capacity of the lung for carbon monoxide. 

Radiographic findings of HPS are nonspecific which include reticular and interstitial opacities, perihilar fibrosis, and pleural thickening [9]. HRCT is the diagnostic imaging of choice for the evaluation of underlying fibrosis and early lung changes. Previous studies have classified the findings on HRCT into multiple grades based on severity 7. Grade 0: normal study; grade 1: minimal reticular disease; grade 2: moderate disease with traction bronchiectasis, peribronchovascular thickening; grade 3: involvement of greater than two-thirds of the lungs with similar findings as grades 1 and 2 [9]. Typical imaging findings include ground glass opacities and reticulations starting in the periphery with progression centrally [7]. There is diffuse involvement with disease progression with traction bronchiectasis, honeycombing, pleural thickening, and peribronchovascular thickening can be seen in the end stages of the disease. 

Like prior descriptions, our patient also had pulmonary parenchymal changes progressing from the peripheral to the central regions with traction bronchiectasis, and pleural and peribronchovascular thickening. Although it has been described that upper lobes are involved in the more severe stages of disease, in our patient upper lobes were involved earlier in the disease process with significant progression over two years. 

Although there is an overlap of imaging findings with other disease processes such as idiopathic pulmonary fibrosis (IPF) and nonspecific interstitial pneumonitis (NSIP), there are distinguishing features. For IPF, basal predominant honeycombing is a characteristic feature and typically presents in older patients [9,10]. For NSIP there is subpleural sparing, often seen in the setting of connective tissue diseases. Typical fibrotic hypersensitivity pneumonitis is mid to upper lung predominant and central predominance. 

Several diagnostic tests are available for HPS in patients with clinical manifestations including pulse oximetry, pulmonary functioning testing, and genetic testing such as next-generation sequencing and platelet electron microscopy. Pulmonary function testing is often performed in the setting of respiratory insufficiency and cirrhosis. Lung biopsy is usually not performed due to the bleeding risk and the presence of imaging findings [6]. Currently, the treatment for HPS-associated ILD is lung transplantation, and no medications are currently approved for the treatment of HPS, although drugs including pirfenidone inhibitors are under clinical trial. 

## Conclusions

Hermansky-Pudlak syndrome (HPS) is a rare autosomal recessive disorder that presents with oculocutaneous albinism, bleeding diathesis, and additional multisystemic manifestations including pulmonary fibrosis. Typical imaging features seen include ground glass opacities, subpleural reticulations, and traction bronchiectasis. The diagnosis of HPS needs to be made through a multidisciplinary approach based on clinical presentation and identification of subtle imaging differences to discern from other forms of interstitial lung disease. Although lung transplant is the mainstay of treatment, several new drug trials are currently ongoing to obtain better treatment options.
